# Cognitive dysfunction in *NF1 *knock-out mice may result from altered vesicular trafficking of APP/DRD3 complex

**DOI:** 10.1186/1471-2202-7-22

**Published:** 2006-03-08

**Authors:** Elizabeth A Donarum, Rebecca F Halperin, Dietrich A Stephan, Vinodh Narayanan

**Affiliations:** 1 Developmental Neurogenetics Laboratory, Barrow Neurological Institute, St Joseph's Hospital and Medical Center, Phoenix AZ, 85013, USA; 2 Neurogenomics Division, Translational Genomics Research Institute, Phoenix AZ, 85004, USA

## Abstract

**Background:**

It has been estimated that more than 50% of patients with Neurofibromatosis type 1 (NF1) have neurobehavioral impairments which include attention deficit/hyperactivity disorder, visual/spatial learning disabilities, and a myriad of other cognitive developmental problems. The biological mechanisms by which *NF1 *gene mutations lead to such cognitive deficits are not well understood, although excessive Ras signaling and increased GABA mediated inhibition have been implicated. It is proposed that the cognitive deficits in NF1 are the result of dysfunctional cellular trafficking and localization of molecules downstream of the primary gene defect.

**Results:**

To elucidate genes involved in the pathogenic process, gene expression analysis was performed comparing the expression profiles in various brain regions for control and *Nf1*^+/- ^heterozygous mice. Gene expression analysis was performed for hippocampal samples dissected from postnatal day 10, 15, and 20 mice utilizing the Affymetrix Mouse Genome chip (Murine 430 2.0). Analysis of expression profiles between *Nf1*^+/-^and wild-type animals was focused on the hippocampus because of previous studies demonstrating alterations in hippocampal LTP in the *Nf1*^+/- ^mice, and the region's importance in visual/spatial learning. Network analysis identified links between neurofibromin and kinesin genes, which were down regulated in the *Nf1*^+/- ^mice at postnatal days 15 and 20.

**Conclusion:**

Through this analysis, it is proposed that neurofibromin forms a binding complex with amyloid precursor protein (APP) and through filamin proteins interacts with a dopamine receptor (Drd3). Though the effects of these interactions are not yet known, this information may provide novel ideas about the pathogenesis of cognitive defects in NF1 and may facilitate the development of novel targeted therapeutic interventions.

## Background

Neurofibromatosis type 1 (also known as von Recklinghausen disease) is an autosomal dominant disorder with a prevalence of 1 in 3500, and is characterized by hyperpigmented skin macules (café au lait spots), iris tumors (Lisch nodules), and benign tumors of nerve cells (neurofibromas) [1]. Other physical complications observed in NF1 patients include optic pathway gliomas, scoliosis, macrocephaly, epilepsy, chronic headaches, bending of the long bones (pseudoarthrosis), and sphenoid wing dysplasia [2]. Cognitive deficits in spatial learning and memory also accompany these more physical manifestations of NF1 [3]. Though mental retardation is not commonly seen in NF1 patients, a high proportion of children afflicted with NF1 show learning disabilities (30 – 65%) [3]. These children perform poorly on tasks requiring developed spatial memory and visual-spatial functioning. Though the cognitive manifestations of NF1 have been characterized, no substantial link between the genetic and cognitive deficits has been formed. In addition, no link has been shown between specific mutations within the causative gene and the degree of physical and mental impairment.

NF1 is caused by a heterozygous loss of function mutation within the *NF1 *gene located on chromosome 17q11.2. The NF1 gene encodes a ubiquitously expressed cytoplasmic protein called neurofibromin. The suspected function of neurofibromin is based on sequence homology to known GTPase Activating Proteins (GAPs) as well as through cell biological and functional studies of mutant neurofibromin [4]. Neurofibromin inactivates Ras (Ras-GTP) by converting it to Ras-GDP. Loss of neurofibromin within a cell would thus result in constitutive activation of the Ras signaling pathway, ultimately resulting in cell growth. Ras signaling has also been implicated in neuronal activity and synaptic plasticity [5].

It has been hypothesized that neurofibromin may also act as a modulator of adenylyl cyclase or may facilitate microtubule binding [5]. Studies in drosophila, cultured murine neurons, and *Nf1*^-/- ^mouse embryos (E12.5) have shown that neurofibromin is necessary for the activation of adenylyl cyclase by pituitary adenylate cyclase activating peptide (PACAP) [6-9]. *Drosophila *models deficient for neurofibromin have also been used to determine if the learning deficits seen within mammalian samples are caused by the developmental abnormalities seen in NF1 or if the cognitive defects are due directly to decreased neurofibromin activity. Heat-shock induced neurofibromin was expressed in adult *NF1*^-/- ^fruit flies, rescuing the learning deficits, indicating that developmental factors are not causing the cognitive deficits [10]. Heat-shock induced cAMP dependent protein kinase (PKA) expression also rescued the learning deficits in adult *NF1*^-/- ^fruit flies, indicating that the cellular defect must be upstream of PKA within the adenylate cyclase signaling pathway [10]. In this *Drosophila *model it is hypothesized that neurofibromin acts as a GAP specific to G-proteins, influencing the interaction between G-proteins and adenylate cyclase [10]. The elucidation of auxiliary functions of neurofibromin can be facilitated by further study of such model organisms containing targeted mutations of the *Nf1 *gene (*Drosophila *and murine systems).

A mouse model of the cognitive deficits associated with Neurofibromatosis type 1 was first developed in 1994 and has since been utilized in the investigation and characterization of the disease [11,12]. The learning deficits seen in the *Nf1*^+/- ^mice include difficulties in spatial learning and decreased hippocampal long-term potentiation (LTP) [5]. Increased levels of GABA-mediated inhibition have been linked to these cognitive deficits within the mouse model and introduction of a GABA_A _receptor antagonist (Picrotoxin) into the knockout mouse system restores normal LTP in the hippocampus [5]. Double knockout mice heterozygous for mutations in both the *Nf1 *and *K-ras *genes (*Nf1*^+/-^/*K-ras*^+/-^) show similar performance on the hidden water maze task as wildtype mice [5]. Inactivating mutations within the *K-ras *gene decrease the level of functional Ras protein within the cells. Observations that the combination of *Nf1 *and *K-ras *mutations in mice results in normal cognitive function support the link between an increase in Ras activity and visual-spatial learning deficits. Ras activity within cells can also be modulated through the introduction of farnesyl-transferase inhibitors. By blocking the post-translational farnesylation of Ras protein in the *Nf1*^+/- ^mutant mice, performance on visual-spatial tasks are comparable to wildtype mice, rescuing the phenotype [5].

The detailed mechanism by which diminished function of neurofibromin protein leads to defects in hippocampal long term potentiation, and subsequent deficits in cognition and learning is not fully understood. Some of the intermediate steps are dependent on gene transcription and new protein synthesis [13]. It is thus appropriate to study the cumulative effect of *Nf1 *gene mutation in the developing hippocampus, and characterize alterations in gene expression profiles in this model system. Here we describe the results of our studies comparing gene expression profiles in the hippocampi of *Nf1*^+/- ^and wild type mice at postnatal ages 10, 15, and 20 days, a time period that is critical for syanptogenesis and synaptic remodeling in the hippocampus. Application of new high-resolution genomic technologies to the *Nf1 *knock-out mouse model may provide new insight into the mechanisms behind the cognitive impairment in humans with Neurofibromatosis type 1.

## Results

**Figure 1:**
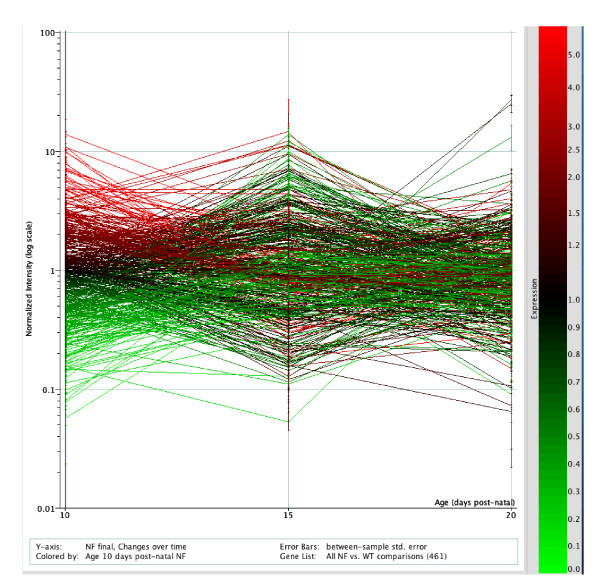
**Visualization of all expression fluctuations in the hippocampus across the time series**. Visualization of genes in the second dataset showing a significant (p ≤ 0.05) fold change of ≥2 between *Nf1*^+/- ^and wild type mice at one or more time points (n = 163). Genes showing increased expression in the mutant model appear in the red portion of the expression color spectrum with decreasing genes in the green portion. The normalized intensity is plotted on a log scale versus the postnatal age. Each line represents the expression of an individual gene.

Genes showing fold change values of ≥2.0 and corresponding p-values of ≤0.05 were visualized across the time series (post natal days 10, 15, and 20) (Fig. 1). Figure 1 shows the expression profiles of genes across the time series and includes only genes which are significantly changed at a minimum of one time point. Individual lists of genes significantly changed at each individual time point are contained in Tables 1, 2, and 3. Four genes were dysregulated at more than one time point: Ate1, Tcfap2d, Rad51l1, Arhgap8. The lists of dysregulated genes include a myriad of genes including enzymes, receptors, channel molecules, and transcription factors. All raw expression data is publicly available [14,15].

RT-PCR validation was performed on a select group of genes showing significantly (p ≤ 0.05) regulated fold changes of ≥2 fold. As can be seen in Table 4, Affymetrix microarray fold change values correlate well with the trend of transcript levels calculated through RT-PCR reactions. Though the exact fold change values are not identical, the two assays show consistent trends of regulation.

Genes significantly dysregulated at post natal days 10, 15, and 20 were entered into the GeneGo network developing program, along with proteins known to be involved in learning and memory (Tab, ErbB-2, CREB, calcium, AMPA, SH2, ShcC, NMDA receptor, TrkB, MAPK, CaM Kinase II, calcineurin, Rho-associated kinase, MAP2, peripherin, ERK1, ERK2, TARP, PAK3) [16]. A functional network was created identifying genes within the data set that are linked to these known mediators of long term potentiation (LTP).

**Figure 2:**
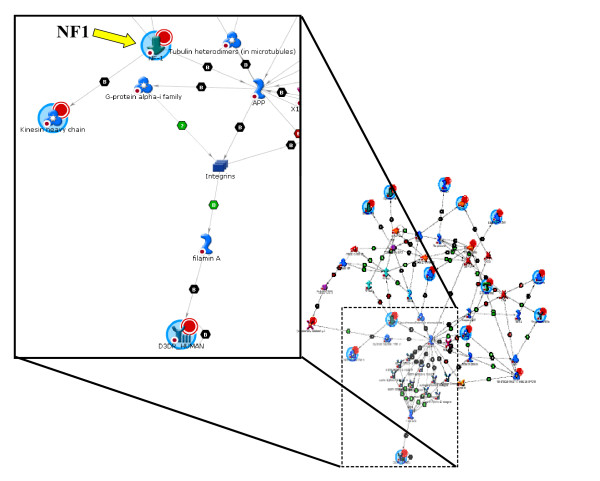
**Clustering network connecting neurofibromin primary gene mutation with downstream cellular factors**. Enlargement of signaling network connecting neurofibromin (Nf-1) with the dopamine 3 receptor (D3DR HUMAN) created through the GeneGo networking software. Neurofibromin is indicated as a binding partner for both the kinesin heavy chain and amyliod beta precursor protein (APP). APP binds through integrins to filamin A, a cytoskeletal organizational protein which in turn can bind to and possibly transport the dopamine 3 receptor.

As expected, the GeneGo networking software identified direct modulation of Ras activity (here notated H-Ras) by neurofibromin. The networking program also identified neurofibromin as a physical binding partner with both the kinesin heavy chain and amyloid beta precursor protein (APP) (Fig. 2). While no significant dysregulation of APP was seen in the data set, members of the kinesin motor protein family were downregulated in the *Nf1*^+/- ^mice at post natal days 15 and 20 (Tables 2 and 3).

Gene expression analysis shows a 2.7 fold increase in the expression of dopamine 3 receptor in *Nf1*^+/- ^brains at post natal day 15. The GeneGo network development software highlights binding properties between this dopamine receptor and filamin A, a protein involved in cytoskeleton organization through binding with integrins, receptors, and second messengers [17]. The associations between integrins and filamin A and between integrins and APP seen in the GeneGo network links neurofibromin to the dopamine receptor. Here it is hypothesized that the APP and integrin proteins are essential for the transport of the dopamine receptor protein down the axon via the filamin proteins. Several other genes linked to intracellular structure and protein trafficking were also dysregulated in the dataset. Aberrant movement of these complexes within the neurons could lead to abnormal localization or abundance of receptors in neuronal processes.

## Discussion

Learning and memory deficits observed in human Neurofibromatosis type 1 patients have been modeled in a *Nf1 *gene knock-out murine system showing well characterized spatial learning and memory deficiencies. These mutant mice exhibit increased levels of activated Ras (Ras-GTP) and increased GABA mediated inhibition. Research has shown that the cognitive deficit in this mouse model can be rescued by inactivating Ras (through genetic modification or pharmacological treatment) or by blocking postsynaptic GABA uptake [5].

We used gene expression profiling to investigate the genetic pathways leading to GABA mediated inhibition, and to link deficiency of neurofibromin to long term changes at the synapse. Differentially regulated genes at postnatal days 10, 15, and 20 were analyzed using GeneGo networking software. This network analysis identified direct interactions between NF1, APP, integrins, filamins, and kinesins. Though compound binding properties were identified *in silico*, these interactions must be investigated within the cells including how these interactions affect the activity of each protein or the localization of the proteins with the cell. It is known that kinesin proteins act within the nerve cell to carry proteins and cellular organelles from the cell body down neuronal processes [18]. Interaction between neurofibromin and kinesins suggests a mechanism for intracellular localization of the neurofibromin/APP complex. Current literature has identified physical interactions between NF1, APP, and kinesin-1 integral to vesicle transport in melanocytes and neurons. This study proposed that NF1 gene mutations impair vesicle trafficking through aberrant kinesin transport of both NF1 and APP [19].

Through network analysis an interaction between APP and the dopamine 3 receptor (DRD3) was idenified. DRD3, a member of the G alpha inhibitory G protein coupled receptor family, was also dysregulated in the mutant mice, showing a 3 fold increase in expression in the hippocampus. The DRD3 receptor is a member of the D2 like dopamine receptor superfamily which selectively mediates inhibition of adenylate cyclase V [20]. The DRD3 receptor expression has been localized to limbic areas of the brain, where it acts via the G_o _subunit and adenylate cyclase to decrease cAMP levels [20,21]. It is unknown if alterations in expression of these receptors are involved in either GABA mediated inhibition, or in other pathways leading to the phenotypic leaning and memory deficits characteristic of NF1.

The results of our network analysis are shown in Figure 2, implying a functional connection between neurofibromin and the amyloid beta precursor protein/integrin/filamin complex, which is in turn related to the dopamine receptors (Drd3). These potential interactions between neurofibromin and APP or DRD3 might lead to new ideas about how neurofibromin is involved in cellular signaling and synaptic plasticity. Future research should include studies of APP and related signaling pathways as well as dopaminergic systems in NF1 models. This also raises the possibility of investigating these pathways in human patients using modern imaging modalities (such as positron emission tomography).

## Methods

### Animals (breeding, dissection, genotyping, and sexing)

*Nf1*^+/- ^mice were purchased from Jackson Laboratory (symbol *Nf1*^*tm*1*Fcr*^) [22]. Breeder pairs were allowed to mate, and offspring were collected at postnatal days 10, 15, and 20. At these ages, mice were euthanized and bilateral brain regions (hippocampus, cerebral cortex, cerebellum, olfactory bulb, and basal ganglion/thalamus) dissected and immediately flash frozen in an ethanol/dry ice bath. Liver and blood were also collected from each mouse. All tissues were stored at -80°C until RNA or DNA extraction was performed.

The *Nf1*^+/- ^mice contain a Neo targeting cassette, which disrupts the *Nf1 *gene to form the knockout allele and can be tested using primers specific for this insert. Genotyping was performed through a series of PCR reactions containing one microliter (approximately 100 ng) of sample DNA, 10 pM of primers, 1× PCR buffer, 2.25 mM MgCl_2_, 10 mM of each dNTP, and 1 unit of Taq Gold polymerase. The PCR cycling program started with 95°C for 5 minutes followed by 35 cycles of 94°C for 1 minute, 55°C for 1 minute, and 72°C for 1 minute. The final step was 72°C for 10 minutes followed by a 4°C hold. Genotyping and sexing primers included:

Control primers (1.2 kilobase product):

mMeC.U256 Forward 5'-GTATGATGACCCCACCTTGC

mMeC.L1452 Reverse 5'-TTCAGTCCCTTCCCGCTTTT

Neo specific primers (2 kilobase product):

Neo5' Forward 5'-GCGTGTTCGAATTCGCCAATG

Exon 32 Reverse 5'-GAAGGACAGCATCAGCATG

Y Chromosome specific primers (200 base pair product):

STS162400 Forward 5'GCAAACAACCTCATAGTCCC

STS162400 Reverse 5'CTGGATTTGTGACAAGGAGC

The reaction product was visualized by 2% agarose gel electrophoresis and the presence of bands noted. The control PCR reaction detected a segment of the MeCP2 gene on the X chromosome, and was used to monitor the integrity of template genomic DNA, and the amplification reaction. The presence of a single 2 kb band in the Neo specific reaction indicated a *Nf1*^+/- ^heterozygous mouse, whereas wild type genomic DNA was represented by absence of a band. Sex was determined by PCR amplification using the Y-chromosome specific primer set. The presence of a band at 200 bp indicates a male mouse and females are shown as the absence of any product.

### Affymetrix expression profiling

Four *Nf1*^+/- ^mice and four age and sex matched wild type mice were analyzed at each time point. Hippocampi (~20 mg each) from two mice within a single condition were pooled and divided to yield two identical samples, and each was individually extracted, labeled, and hybridized to the Affymetrix (Murine 430 2.0) chip. Total RNA was isolated from each 40 mg tissue sample using Stratagene RTPCR Mini-prep kit (the average yield was 15 μg RNA/40 mg tissue). Extracted RNA was subsequently cleaned using the Qiagen Mini kit protocol, and the purified RNA was analyzed through agarose gel electrophoresis to insure quality.

cDNA was synthesis from 7 μg of purified total RNA, *in vitro *transcription, and hybridization proceeded as previously described [21]. Strict quality controls require that each RNA sample show >4 × amplification through the *in vitro *transcription protocol, that each scanned array should contain >30% present calls across the array, and that the 3'/5' should show consistent values >3 indicating low nonspecific hybridization. Arrays that do not satisfy these conditions were not included in the analysis and a second sample of cRNA was created utilizing a second allotment of stored total RNA from the sample.

### Data analysis

Data was extracted from the array images using Affymetrix Microarray Suite version 5.1 software (MAS5.0). Raw expression data was corrected for saturation at individual probes using an in-house Array Data Manipulation program which replaces S2 values with S1 values if the S2 values are greater than 1500 (baseline normalization of 150) or the S2/S1 signal ratio is less than 0.8.

The modified gene expression data for each individual array was imported into GeneSpring v 5.0 (Agilent Technologies). For each time point, average fold changes (relative to wildtype expression data) were calculated with error bars. Genes showing expression changes with significant p-values (p ≤ 0.05) and fold change values of ≥2.0 within at least one time point were exported for functional annotation. Thereafter, the function of each gene was determined through literature searches, genes were binned into ontologic categories, and relevant biological processes and pathways identified.

### Modeling the dysfunctional genetic network

The main goal of both temporal and functional clustering is to generate an integrated pathway beginning with the known primary genetic defect and ending with proteins known to be involved in causing the cognitive pathology under study. This pathway then becomes the template for later *in vivo *validation. The GeneGo network building algorithms (GeneGo, Inc) were used in an iterative fashion to build gene/protein interaction pathways between known NF1 pathway members (NF1, Ras, GABA) and proteins known to be involved in LTP. The gene expression changes with ≥2 fold differences at p ≤ 0.05 were used to seed the algorithms and identified new pathway members which link the primary defect to the cognitive phenotype. All raw expression data is publicly available [14,15].

### Validation of the pathogenic cascade

*Quantitative Real-Time PCR *– Total RNA was extracted from ~20 mg of hippocampus from 3 *Nf1*^+/- ^and 3 wild type mice using the Absolutely RNA Miniprep Kit (Stratagene). Reverse transcription reactions were done using 3 μg of total RNA from hippocampus, oligo dT primers, and the Super Script III First Strand cDNA synthesis kit (Invitrogen). Resulting cDNA was amplified on the Chromo4 Four-Color Real-Time System (MJ Research) using the DyNAmo HS SYBR Green qPCR Kit (Finnzymes) and gene specific primers. Standardized and optimized primers were ordered from SuperArray Bioscience Corporation. These included primers designed for Stc1 (stanniocalcin1), Htr5a (5-hydroxytryptamine (serotonin) receptor 5A), Neto2 (neuropilin and tolloid like protein 2), and Frap1 (FK506 binding protein 12-rapamycin associated protein1). The housekeeping gene GAPD (glyceraldehydes-3-phosphate dehydrogenase) was analyzed using the primer set (f-CCAGTATGACTCCACTCACG, r-GAGATGATGACCCGTTTGGC). For amplification, the following program was employed: a 95°C heat activation step for 15 min, followed by 40 cycles of 94°C for 10 sec, 55°C for 25 sec, 72°C for 30 sec, incubate at 72°C, and plate reads at both 77°C and 81°C. A melting curve was created evaluating the products between 60–95°C reading every 0.2°C.

Primer set specificity was verified through melting curve analysis. The threshold for amplification was set as the number of cycles necessary to reach logarithmic fluorescence accumulation (C(T)). Fold difference in cDNA concentration was calculated using the formula F = 2^((MH-MG)-(WH-WG)) ^where F = fold difference, MH = mutant housekeeping gene (GAPD) C(T), MG = mutant gene of interest C(T), WH = wild type housekeeping gene (GAPD) C(T), WG = wild type gene of interest C(T) [24,25]. Statistical significance of the resulting fold change values was calculated with a two-tailed t-test assuming unequal variance.

## Authors' contributions

EAD performed all animal breeding and dissection, as well as network analysis and RT-PCR validation. RH performed all Affymetrix expression profiling. DAS developed experimental design and participated in network and data analysis. VN conceived of the study and assisted in data analysis and interpretation of results. All authors read and approved the final manuscript.

## Figures and Tables

**Table 1:** Genes at post natal day 10 showing fold change values of ≥2 between *Nf1*^+/- ^and wild type mice (p ≤ 0.05).

Probe Set	Unigene	Fold Change	P Value	Gene Name	Gene Symbol
1421984_at	Mm.20911	12.2	0.00205	stanniocalcin 1	Stc1
1422207_at	Mm.4835	9.391	0.000624	5-hydroxytryptamine (serotonin) receptor 5A	Htr5a
1426141_at	Mm.207059	7.518	0.0351	RF-amide G protein-coupled receptor	MrgA1
1445983_at	Mm.172835	6.707	0.0139	ubiquitin-conjugating enzyme E2A, RAD6 homolog	Ube2a
1436615_a_at	Mm.2611	6.396	0.0416	ornithine transcarbamylase	Otc
1457713_at	Mm.2213	6.388	0.0281	excision repair cross-complementing rodent repair deficiency	Ercc5
1459730_at	Mm.52297	5.653	0.0329	formin binding protein 1	Fnbp1
1446315_at	Mm.282039	5.376	0.0362	ATP citrate lyase	Acly
1430486_at	Mm.341756	4.883	0.0493	RAD51-like 1 (S. cerevisiae)	Rad51l1
1425443_at	Mm.244858	4.441	0.0239	transcription factor AP-2, delta	Tcfap2d
1452431_s_at	Mm.235338	4.175	0.0242	histocompatibility 2, class II antigen A, alpha	H2-Aa
1433133_at	Mm.159671	3.708	0.0269	EDAR (ectodysplasin-A receptor)-associated death domain	Edaradd
1429566_a_at	Mm.23790	3.69	0.0369	homeodomain interacting protein kinase 2	Hipk2
1426005_at	Mm.199008	3.673	0.00896	dentin matrix protein 1	Dmp1
1429668_at	Mm.246550	3.67	0.0389	POU domain, class 4, transcription factor 1	Pou4f1
1415861_at	Mm.30438	3.651	0.0382	tyrosinase-related protein 1	Tyrp1
1427313_at	Mm.287572	3.557	0.0125	prostaglandin I receptor (IP)	Ptgir
1435663_at	Mm.9213	3.449	0.00753	estrogen receptor 1 (alpha)	Esr1
1441957_x_at	Mm.205196	3.211	0.0202	stromal cell derived factor receptor 1	Sdfr1
1422038_a_at	Mm.261384	3.19	0.0384	tumor necrosis factor receptor superfamily, member 22	Tnfrsf22
1421967_at	Mm.200886	3.031	0.0318	UDP-Gal:betaGlcNAc beta 1,4-galactosyltransferase, polypeptide 5	B4galt5
1419085_at	Mm.41456	2.949	0.0459	Purkinje cell protein 2 (L7)	Pcp2
1456151_at	Mm.299073	2.931	0.0198	zinc finger protein 358	Zfp358
1449484_at	Mm.32506	2.929	0.00956	stanniocalcin 2	Stc2
1436490_x_at	Mm.297440	2.888	0.0446	RAN, member RAS oncogene family	Ran
1447786_at	Mm.86413	2.684	0.0443	pleckstrin homology, Sec7 and coiled-coil domains 1	Pscd1
1457684_at	Mm.277465	2.683	0.0331	heat shock protein 12B	Hspa12b
1437571_at	Mm.261602	2.572	0.023	hypermethylated in cancer 2	Hic2
1427637_a_at	Mm.89935	2.516	0.048	desmocollin 3	Dsc3
1421275_s_at	Mm.354761	2.438	0.00644	suppressor of cytokine signaling 7	Socs7
1444641_at	Mm.71996	2.351	0.0412	adenylate cyclase 3	Adcy3
1459828_at	Mm.1963	2.326	0.00118	serine/arginine repetitive matrix 1	Srrm1
1421660_at	Mm.314531	2.323	0.026	sodium channel, voltage-gated, type IX, alpha polypeptide	Scn9a
1445314_at	Mm.4866	2.302	0.000693	ets variant gene 1	Etv1
1421234_at	Mm.332607	2.248	0.0373	transcription factor 1	Tcf1
1442456_at	Mm.172679	2.215	0.0262	spermatogenesis associated 5	Spata5
1441483_at	Mm.336081	2.153	0.0398	SLIT and NTRK-like family, member 2	Slitrk2
1438597_x_at	Mm.196508	2.137	0.0365	mortality factor 4 like 1	Morf4l1
1439428_x_at	Mm.247143	2.118	0.0407	GDP-mannose 4, 6-dehydratase	Gmds
1459924_at	Mm.340818	2.088	0.0306	ATPase, H+ transporting, lysosomal V0 subunit a isoform 1	Atp6v0a1
1425429_s_at	Mm.354757	2.003	0.048	hypoxia inducible factor 3, alpha subunit	Hif3a
1427040_at	Mm.1314	0.499	0.0418	kidney cell line derived transcript 1	Kdt1
1418745_at	Mm.139817	0.498	0.0139	osteomodulin	Omd
1419230_at	Mm.342959	0.49	0.00148	keratin complex 1, acidic, gene 12	Krt1-12
1423170_at	Mm.236009	0.487	0.000269	TAF7 RNA polymerase II, TATA box binding protein (TBP)-associated factor	Taf7
1419231_s_at	Mm.342959	0.487	0.00492	keratin complex 1, acidic, gene 12	Krt1-12
1457306_at	Mm.6988	0.483	0.0387	aminolevulinate, delta-, dehydratase	Alad
1432834_at	Mm.24242	0.475	0.00679	carboxypeptidase B2 (plasma)	Cpb2
1449739_at	Mm.281464	0.472	0.0126	phosphatidylserine synthase 1	Ptdss1
1460250_at	Mm.43375	0.466	0.00542	sclerostin domain containing 1	Sostdc1
1425291_at	Mm.4985	0.466	0.00137	forkhead box J1	Foxj1
1442344_at	Mm.82680	0.465	0.00971	AP1 gamma subunit binding protein 1	Ap1gbp1
1456247_x_at	Mm.18565	0.462	0.0237	LIM domain only 6	Lmo6
1450760_a_at	Mm.39999	0.462	0.0157	inhibitor of growth family, member 3	Ing3
1418082_at	Mm.10265	0.456	0.0305	N-myristoyltransferase 1	Nmt1
1427612_at	Mm.171224	0.444	0.00697	defensin beta 9	Defb9
1439051_a_at	Mm.260504	0.442	0.0497	MAP/microtubule affinity-regulating kinase 4	Mark4
1421906_at	Mm.12926	0.427	0.0224	peroxisome proliferator activated receptor binding protein	Pparbp
1416203_at	Mm.18625	0.427	0.0261	aquaporin 1	Aqp1
1444960_at	Mm.243855	0.412	0.031	cytochrome P450, family 2, subfamily u, polypeptide 1	Cyp2u1
1424273_at	Mm.29119	0.402	0.00623	cytochrome P450, family 2, subfamily c, polypeptide 70	Cyp2c70
1450995_at	Mm.2135	0.37	0.0443	folate receptor 1 (adult)	Folr1
1418554_at	Mm.2857	0.366	0.0107	adrenomedullin receptor	Admr
1424713_at	Mm.28623	0.36	0.0295	calmodulin-like 4	Calml4
1454866_s_at	Mm.44747	0.357	0.00964	chloride intracellular channel 6	Clic6
1449693_at	Mm.258589	0.353	0.0446	mitogen activated protein kinase kinase kinase 7	Map3k7
1452546_x_at	Mm.221026	0.337	0.0323	defensin beta 11	Defb11
1419662_at	Mm.4258	0.332	0.0234	osteoglycin	Ogn
1417297_at	Mm.328900	0.294	0.0313	inositol 1,4,5-triphosphate receptor 3	Itpr3
1436477_x_at	Mm.240224	0.288	0.0103	RAB2, member RAS oncogene family	Rab2
1459738_x_at	Mm.1114	0.286	0.0425	galactosidase, alpha	Gla
1439167_at	Mm.281738	0.284	0.0116	peroxisomal trans-2-enoyl-CoA reductase	Pecr
1420652_at	Mm.216321	0.27	0.0445	arginine-tRNA-protein transferase 1	Ate1
1427560_at	Mm.3410	0.259	0.0328	sine oculis-related homeobox 5 homolog (Drosophila)	Six5
1435214_at	Mm.40016	0.241	0.00726	gap junction membrane channel protein alpha 12	Gja12
1425794_at	Mm.209931	0.236	0.0234	polymerase (DNA directed), alpha 2	Pola2
1426151_a_at	Mm.272264	0.235	0.0214	syntaxin 3	Stx3
1439878_at	Mm.207365	0.208	0.0151	involucrin	Ivl
1450805_at	Mm.338890	0.202	0.0179	sarcoglycan, delta (dystrophin-associated glycoprotein)	Sgcd
1438406_at	Mm.194950	0.195	0.00559	scavenger receptor class F, member 2	Scarf2
1439457_x_at	Mm.9852	0.189	0.0278	autophagy 12-like (S. cerevisiae)	Apg12l
1444680_at	Mm.208970	0.171	0.0137	positive cofactor 2, multiprotein complex, glutamine/Q-rich-associated protein	Pcqap
1426171_x_at	Mm.193478	0.17	0.0166	killer cell lectin-like receptor, subfamily A, member 7	Klra7
1418618_at	Mm.2657	0.0828	6.20E-05	engrailed 1	En1

**Table 2:** Genes at post natal day 15 showing fold change values of ≥2 between *Nf1*^+/- ^and wild type mice (p ≤ 0.05)

Probe Set	Unigene	Fold Change	P Value	Gene Name	Gene Symbol
1425754_a_at	Mm.1420	12.45	0.0354	butyrophilin, subfamily 1, member A1	Btn1a1
1418555_x_at	Mm.21642	6.649	0.00469	Spi-C transcription factor (Spi-1/PU.1 related)	Spic
1420992_at	Mm.10279	6.523	0.00774	ankyrin repeat domain 1 (cardiac muscle)	Ankrd1
1418358_at	Mm.331192	6.182	0.0229	mitochondrial capsule selenoprotein	Mcsp
1458958_at	Mm.209041	6.01	0.00798	neighbor of Punc E11	Nope
1447392_s_at	Mm.276736	5.357	0.0228	carboxypeptidase D	Cpd
1450439_at	Mm.248353	5.224	0.0198	host cell factor C1	Hcfc1
1435410_at	Mm.159608	4.96	0.0419	testicular cell adhesion molecule 1	Tcam1
1454032_at	Mm.126079	4.831	0.0386	neuropilin (NRP) and tolloid (TLL)-like 2	Neto2
1438414_at	Mm.39703	4.337	0.0381	fukutin related protein	Fkrp
1420652_at	Mm.216321	4.245	0.0175	arginine-tRNA-protein transferase 1	Ate1
1425069_at	Mm.264252	4.15	0.00663	similar to nuclear protein, 25 K – mouse	LOC223706
1421515_at	Mm.242728	3.998	0.0161	nuclear receptor subfamily 6, group A, member 1	Nr6a1
1447362_at	Mm.29133	3.983	0.04	budding uninhibited by benzimidazoles 1 homolog, beta	Bub1b
1456697_x_at	Mm.22480	3.814	0.0171	cyclin D binding myb-like transcription factor 1	Dmtf1
1422260_x_at	Mm.14302	3.75	0.0144	chemokine (C-C motif) receptor 5	Ccr5
1420253_at	Mm.201322	3.638	0.0362	dolichol-phosphate (beta-D) mannosyltransferase 1	Dpm1
1427825_at	Mm.272223	3.568	0.0202	solute carrier organic anion transporter family, member 1b2	Slco1b2
1458678_at	Mm.347976	3.356	0.00654	NADH dehydrogenase (ubiquinone) 1, alpha/beta subcomplex, 1	Ndufab1
1441659_at	Mm.151308	3.305	0.0274	D4, zinc and double PHD fingers, family 3	Dpf3
1417015_at	Mm.41265	3.094	0.0256	Ras association (RalGDS/AF-6) domain family 3	Rassf3
1418536_at	Mm.34421	3.034	0.0147	histocompatibility 2, Q region locus 7	H2-Q7
1437847_x_at	Mm.28275	2.999	0.0357	RNA binding motif protein, X chromosome	Rbmx
1440837_at	Mm.358604	2.771	0.031	histocompatibility 2, O region beta locus	H2-Ob
1418595_at	Mm.12966	2.751	0.041	plasma membrane associated protein, S3-12	S3-12
1422278_at	Mm.327835	2.707	0.00471	dopamine receptor 3	Drd3
1425398_at	Mm.371766	2.539	0.0134	histone 1, H2bc	Hist1h2bc
1425443_at	Mm.244858	2.53	0.0347	transcription factor AP-2, delta	Tcfap2d
1419623_at	Mm.86657	2.515	0.0308	protease, serine, 21	Prss21
1421359_at	Mm.57199	2.51	0.000164	ret proto-oncogene	Ret
1450455_s_at	Mm.89993	2.504	0.00985	aldo-keto reductase family 1, member C12	Akr1c12
1420710_at	Mm.4869	2.422	0.0149	reticuloendotheliosis oncogene	Rel
1434885_at	Mm.155687	2.357	0.0457	DNA segment, Chr 7, ERATO Doi 413, expressed	D7Ertd413e
1451463_at	Mm.291372	2.351	0.0344	Rho GTPase activating protein 8	Arhgap8
1416309_at	Mm.290015	2.299	0.00369	nucleolar and spindle associated protein 1	Nusap1
1425064_at	Mm.250265	2.272	0.00144	aryl hydrocarbon receptor nuclear translocator	Arnt
1425721_at	Mm.221688	2.245	0.0475	pleckstrin homology domain interacting protein	Phip
1421953_at	Mm.21048	2.219	0.0017	v-crk sarcoma virus CT10 oncogene homolog (avian)-like	Crkl
1426520_at	Mm.104932	2.199	0.0211	B-cell translocation gene 4	Btg4
1450104_at	Mm.3037	2.172	0.0339	a disintegrin and metalloprotease domain 10	Adam10
1436008_at	Mm.371590	2.134	0.00836	tumor protein D52	Tpd52
1419535_at	Mm.263706	2.121	0.0243	solute carrier organic anion transporter family, member 6b1	Slco6b1
1420499_at	Mm.10651	2.121	0.0318	GTP cyclohydrolase 1	Gch
1451870_a_at	Mm.253518	2.011	0.0256	bromodomain containing 4	Brd4
1445886_at	Mm.4454	0.497	0.0367	ELK3, member of ETS oncogene family	Elk3
1425690_at	Mm.218788	0.461	0.0463	beta-1,3-glucuronyltransferase 1 (glucuronosyltransferase P)	B3gat1
1441966_at	Mm.124567	0.452	0.0279	transient receptor potential cation channel, subfamily M, member 3	Trpm3
1427635_at	Mm.30355	0.438	0.0348	kinesin family member 5A	Kif5a
1418194_at	Mm.271670	0.421	0.0445	UDP-N-acetyl-alpha-D-galactosamine:polypeptide N-acetylgalactosaminyltransferase 10	Galnt10
1416239_at	Mm.3217	0.42	0.00011	argininosuccinate synthetase 1	Ass1
1449266_at	Mm.131408	0.415	0.0128	methyl CpG binding protein 2	Mecp2
1440072_at	Mm.210787	0.407	0.021	glucocorticoid induced transcript 1	Glcci1
1430357_at	Mm.371563	0.398	0.0139	H3 histone, family 3B	H3f3b
1425707_a_at	Mm.328720	0.387	0.0192	potassium inwardly-rectifying channel, subfamily J, member 6	Kcnj6
1446185_at	Mm.21158	0.353	0.0479	FK506 binding protein 12-rapamycin associated protein 1	Frap1
1422144_at	Mm.3510	0.328	0.0259	inhibin beta E	Inhbe
1421447_at	Mm.303355	0.298	0.000377	one cut domain, family member 1	Onecut1
1449907_at	Mm.174133	0.255	0.0278	beta-carotene 15, 15'-dioxygenase 1	Bcdo1
1421073_a_at	Mm.18509	0.237	0.0359	prostaglandin E receptor 4 (subtype EP4)	Ptger4
1420296_at	Mm.254370	0.232	0.00578	chloride channel 5	Clcn5
1418158_at	Mm.20894	0.205	0.0342	transformation related protein 63	Trp63
1448074_at	Mm.5033	0.174	0.0361	relaxin 1	Rln1
1453630_at	Mm.271953	0.122	0.00471	UDP-N-acetyl-alpha-D-galactosamine:polypeptide N-acetylgalactosaminyltransferase 14	Galnt14
1446162_at	Mm.125979	0.0938	0.0101	poly A binding protein, cytoplasmic 5	Pabpc5

**Table 3:** Genes at post natal day 20 showing fold change values of ≥2 between *Nf1*^+/- ^and wild type mice (p ≤ 0.05)

Probe Set	Unigene	Fold Change	P Value	Gene Name	Gene Symbol
1425947_at	Mm.240327	13.26	0.000122	interferon gamma	Ifng
1442827_at	Mm.38049	4.568	0.0446	toll-like receptor 4	Tlr4
1425478_x_at	Mm.240044	4.347	0.0178	ubiquitin-conjugating enzyme E2I	Ube2i
1450337_a_at	Mm.23788	3.618	0.0132	NIMA (never in mitosis gene a)-related expressed kinase 8	Nek8
1422411_s_at	Mm.327088	3.478	0.0376	ribonuclease, RNase A family 3	Rnase3
1422297_at	Mm.158264	2.727	0.0381	prefoldin 5	Pfdn5
1438564_at	Mm.207360	2.573	0.0405	growth arrest specific 2	Gas2
1433732_x_at	Mm.281018	2.313	0.0229	insulin-like growth factor 2, binding protein 3	Igf2bp3
1427816_at	Mm.21841	2.306	0.0137	splicing factor, arginine/serine-rich 2 (SC-35)	Sfrs2
1452349_x_at	Mm.255414	2.149	0.0043	interferon activated gene 205	Ifi205
1418604_at	Mm.4351	2.038	0.0134	arginine vasopressin receptor 1A	Avpr1a
1438156_x_at	Mm.18522	0.496	0.0254	carnitine palmitoyltransferase 1a, liver	Cpt1a
1426714_at	Mm.131618	0.488	0.00577	DNA segment, Chr 11, ERATO Doi 18	D11Ertd18e
1455332_x_at	Mm.330161	0.48	0.00869	Fc receptor, IgG, low affinity IIb	Fcgr2b
1450951_at	Mm.14910	0.478	0.0373	chondroitin sulfate proteoglycan 6	Cspg6
1423719_at	Mm.3783	0.359	0.0127	cDNA sequence U46068	U46068
1419109_at	Mm.39968	0.357	0.0432	histidine rich calcium binding protein	Hrc
1460746_at	Mm.236114	0.356	0.0339	fidgetin-like 1	Fignl1
1433382_at	Mm.79127	0.352	0.0427	dynein, axonemal, intermediate chain 1	Dnaic1
1449207_a_at	Mm.258846	0.297	0.0232	kinesin family member 20A	Kif20a
1455990_at	Mm.259374	0.279	0.00332	kinesin family member 23	Kif23
1436682_at	Mm.3532	0.273	0.0428	thymosin, beta 10	Tmsb10
1430486_at	Mm.341756	0.232	0.0143	RAD51-like 1 (S. cerevisiae)	Rad51l1
1426598_at	Mm.20477	0.222	0.0128	ubiquitously transcribed tetratricopeptide repeat gene, Y chromosome	Uty
1451463_at	Mm.291372	0.193	0.0173	Rho GTPase activating protein 8	Arhgap8
1441429_at	Mm.261591	0.188	0.036	insulin receptor substrate 4	Irs4
1452563_a_at	Mm.262676	0.109	0.00609	selected mouse cDNA on the Y	Smcy
1457582_at	Mm.20477	0.0739	0.0209	ubiquitously transcribed tetratricopeptide repeat gene, Y chromosome	Uty
1426438_at	Mm.302938	0.0533	0.0444	DEAD (Asp-Glu-Ala-Asp) box polypeptide 3, Y-linked	Ddx3y

**Table 4:** Affymetrix and RT-PCR fold change values for genes significantly regulated in the hippocampus of *Nf1*^+/- ^mice

Gene Symbol	Gene Name	Probe ID	Fold Change	P-value	RT-PCR Fold Change	RT-PCR P-value
Stc1	stanniocalcin 1	1421984_at	12.2	0.0021	1.7592	0.0100
Htr5a	5-hydroxytryptamine (serotonin) receptor 5A	1422207_at	9.391	0.0006	2.0005	0.0580
Neto2	neuropilin (NRP) and tolloid (TLL)-like 2	1454032_at	4.831	0.0386	1.4045	0.0415
Frap1	FK506 binding protein12-rapamycin associated protein 1	1446185_at	0.353	0.0479	0.3927	0.4711
